# A transcriptomic dataset used to derive biomarkers of chemically induced histone deacetylase inhibition (HDACi) in human TK6 cells

**DOI:** 10.1016/j.dib.2021.107097

**Published:** 2021-04-29

**Authors:** Eunnara Cho, Andrew Williams, Carole L. Yauk

**Affiliations:** a Environmental Health Science and Research Bureau, Health Canada, Ottawa, ON, Canada; b Department of Biology, Carleton University, Ottawa, ON, Canada; c Department of Biology, University of Ottawa, Ottawa, ON, Canada

**Keywords:** Transcriptomic biomarker, Toxicogenomics, Predictive toxicology, Histone deacetylase inhibition, TempO-Seq, Epigenetics, Genomics

## Abstract

Transcriptomic biomarkers facilitate mode of action analysis of toxicants by detecting specific patterns of gene expression perturbations. We identified an 81-gene transcriptomic biomarker of histone deacetylase inhibitors (HDACi) using whole transcriptome data sets of TK6 human lymphoblastoid cells generated by Templated Oligo-Sequencing (TempO-Seq) after 4 h of exposure to 20 reference compounds (10 HDACi and 10 non-HDACi) [1]. The biomarker, named TGx-HDACi, was derived using the nearest shrunken centroid (NSC) method and can distinguish HDACi from non-HDACi compounds based on the expression pattern across the 81 genes. The classification capability of TGx-HDACi was evaluated by NSC probability analysis of 11 external validation compounds (4 HDACi and 7 non-HDACi) with a probability cut-off of 90%. Thus far, TGx-HDACi has demonstrated 100% accuracy in classifying the reference and validation compounds as HDACi or non-HDACi. Of the 81 TGx-HDACi genes, 19 genes are part of the S1500+ gene panel containing 2753 genes, developed for toxicological assessments [2]. Herein, we assessed the classification performance of the biomarker with this reduced gene set to determine if TGx-HDACi can be applied to analyze S1500+ gene expression profiles. The 20 reference compounds and 11 validation compounds were correctly classified as HDACi or non-HDACi by the NSC probability analysis, principal component analysis, and hierarchical clustering based on the expression of the 19 genes, demonstrating 100% accuracy.

## Specifications Table

**Table:** 

Subject	Omics: Toxicogenomics (TGx)
Specific subject area	TGx is the study of genomic responses to toxicity. Transcriptomic biomarkers are patterns of gene expression changes that predict specific toxicities.
Type of data	Transcriptomic data Tables Figures
How data were acquired	Whole transcriptome Templated Oligo-Sequencing (TempO-Seq) (BioSpyder, Carlsbad, CA, USA) libraries were sequenced on an Illumina NextSeq 500 sequencer using 75-cycle flow cells.
Data format	All raw data (i.e., FASTQ files) and normalized transcriptomic data (TXT file containing transcript read counts normalized to counts per million) have been deposited to the National Center for Biotechnology Information (NCBI)’s Gene Expression Omnibus (GEO) database, under the accession number GSE164478. The overall centroids of the 81 TGx-HDACi genes derived from the TempO-Seq whole transcriptome profiles of the 20 reference compounds are presented in Supplementary Table 1. The overall centroids of 19 genes used in the analysis herein are presented within this article in Table 1. Analyses of the data are presented as figures.
Parameters for data collection	TK6 human lymphoblastoid cells were exposed to 31 compounds (HDACi and non-HDACi) and the vehicle solvents for 4 hr. The concentration of each compound was selected based on cell viability (>60%) at 24 hr and fold changes in three select genes measured in a preliminary qPCR experiment. There were three replicates and solvent matched controls for each chemical.
Description of data collection	Using total RNA extracted from TK6 cells, whole transcriptome TempO-Seq libraries were constructed. The libraries were sequenced on an Illumina NextSeq 500 sequencer. The resulting BCL files were converted to FASTQ files, which were processed to align the reads and generate counts for each gene. The read counts of all samples were normalized to counts per million (CPM) and the CPM values of each chemical treatment was then normalized to its solvent control. The nearest shrunken centroid method was applied to the resulting gene expression profiles to derive an 81-gene biomarker of HDACi.
Data accessibility	Data are partly hosted with the article and the complete TempO-Seq dataset is available in a public repository. Repository name: Gene Expression Omnibus (GEO) Data identification number: GSE164478 Direct URL to data: https://www.ncbi.nlm.nih.gov/geo/query/acc.cgi?acc=GSE164478
Related research article	E. Cho, A. Rowan-Carroll, A. Williams, J.C. Corton, H.H. Li, A. Fornace Jr., C. Hobb, C.L. Yauk, Development and Validation of the TGx-HDACi Transcriptomic Biomarker to Detect Histone Deacetylase Inhibitors in Human TK6 Cells, Arch Toxicol. (2021) https://doi.org/10.1007/s00204-021-03014-2.

## Value of the Data

• These data demonstrate that TGx-HDACi, an 81-gene biomarker of HDACi, retains its ability to accurately classify chemicals when applied using a subset of 19 genes of the biomarker that are part of the S1500+ gene panel for toxicological analysis; this gene panel is commercially available for use on the TempO-Seq transcriptomics platform.
• The training set data and the 81- or 19-gene biomarker can be applied by researchers to efficiently analyze new and existing transcriptomic profiles of cultured human cells exposed to chemicals to identify potential HDACi agents and conditions.
• The biomarker can be used to screen existing gene expression profiles of chemicals in databases to identify potential HDACi compounds and to analyze new gene expression data; the reduced gene set facilitates TGx-HDACi analysis of non-whole transcriptome gene expression profiles, which may not contain all 81 genes.
• In addition, the complete TempO-Seq dataset from which TGx-HDACi was derived and validated contains whole transcriptome profiles of 31 compounds of diverse modes of action; these data could be leveraged to develop additional transcriptomic biomarkers to diversify the modes of action that can be detected in gene expression profiles of TK6 cells.

## 1 Data Description

Whole transcriptome expression profiles were generated by TempO-Seq in TK6 cells exposed to HDACi compounds and non-HDACi compounds of diverse mechanisms for 4 h (GSE164478). This dataset contains raw data files (FASTQ files) and the normalized transcriptomic profiles containing CPM values of the TGx-HDACi biomarker reference set (10 HDACi and 10 non-HDACi compounds) and the external validation set (4 HDACi and 7 non-HDACi). The list of compounds is presented Table 1. The concentration used for each compound is included in Cho et al. (2021).

**Table 1:** List of reference and validation compounds with chemical class and abbreviations.

	Class	Chemical	Abbreviation
Reference Compound	HDACi	Apicidin	Api
		Mocetinostat	Mo
		Oxamflatin	Oxa
		Panobinostat	Pan
		Scriptaid	Scr
		Sodium butyrate	SodButy
		Suberohydroxamic acid	SBHA
		Tacedinaline	Tac
		Trichostatin A	TSA
		Vorinostat	Vor

	Non-HDACi	2-Deoxyglucose	2-DG
		5-Fluorouracil	5-FU
		Antimycin A	Ant
		Bleomycin	Ble
		Cadmium chloride	CdCl_2_
		Cisplatin	Cisp
		Camptothecin	CPT
		Hydroxyurea	HU
		Tunicamycin	Tun
	Vinblastine	Vin

External Validation Compound	HDACi	Entinostat	Ent
		HC Toxin	HCT
		Pracinostat	Pra
		Valproic acid	VPA

	Non-HDACi	Arabinofuranosyl cytidine	AraC
		Docetaxel	Doc
		Methotrexate	MTX
		Thapsigargin	Thap
		3-Deazaneplanocin A	DZNep
		Garcinol	Gar
	GSK-J4	GSK-J4

The nearest shrunken centroid (NSC) method was applied to the TGx-HDACi reference gene expression profiles to derive a panel of 81 genes whose expression pattern can distinguish between the two chemical classes; the shrunken centroids of the 81-gene biomarker are included in Supplementary Table 1. Table 2 presents the shrunken centroids of 19 genes that are part of both the TGx-HDACi biomarker and the S1500+ gene set.

**Table 2:** The centroids and standard deviations of 19 genes from the TGx-HDACi biomarker.

Gene	HDACi Class Centroid	Non-HDACi Class Centroid	Standard Deviation
*BMF*	3.564	0.135	1.171
*ID1*	2.287	−2.029	2.607
*MYBL1*	2.279	0.344	1.122
*DYRK3*	1.769	−0.031	0.860
*IFI6*	1.506	0.044	0.896
*POTEM*	1.274	−0.220	0.920
*ATP1B1*	1.268	−0.645	0.898
*ST3GAL5*	1.257	−0.414	0.906
*TUBB2A*	1.243	−0.358	1.017
*TMEM2*	1.237	−0.465	1.031

*FAS*	−1.317	0.353	1.007
*E2F8*	−1.497	0.226	0.985
*CDK5R1*	−1.542	0.043	0.954
*RIPK1*	−1.615	0.346	1.227
*PPIL1*	−1.709	0.078	0.978
*AKAP8*	−1.758	−0.086	0.930
*COIL*	−1.794	0.191	1.073
*SUV39H1*	−1.867	−0.003	1.102
*GPR183*	−2.712	−0.279	1.273

**Fig. 1:**
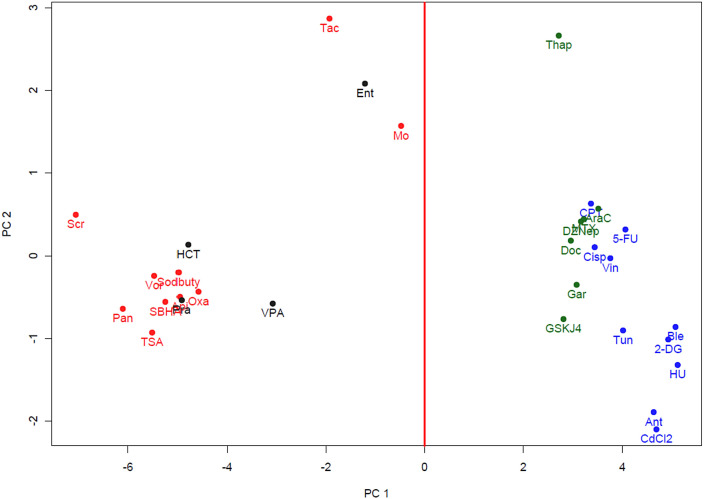
Principal component analysis of the 19-gene expression profiles in TK6 cells exposed to the 20 TGx-HDACi reference compounds and 11 external validation compounds for 4 h. The red dots represent the 10 HDACi reference compounds and the blue dots represent the 10 non-HDACi reference compounds. Each dot represents the average of three replicates. The vertical red line at principal component 1 (PC1) separates HDACi from non-HDACi for classification purposes. PC1 describes the contrast between the HDACi and the non-HDACi classes. The four HDACi external validation compounds are in black and the seven non-HDACi validation compounds are in green; all validation compounds clustered within their respective classes (For interpretation of the references to color in this figure legend, the reader is referred to the web version of this article.).

**Fig. 2:**
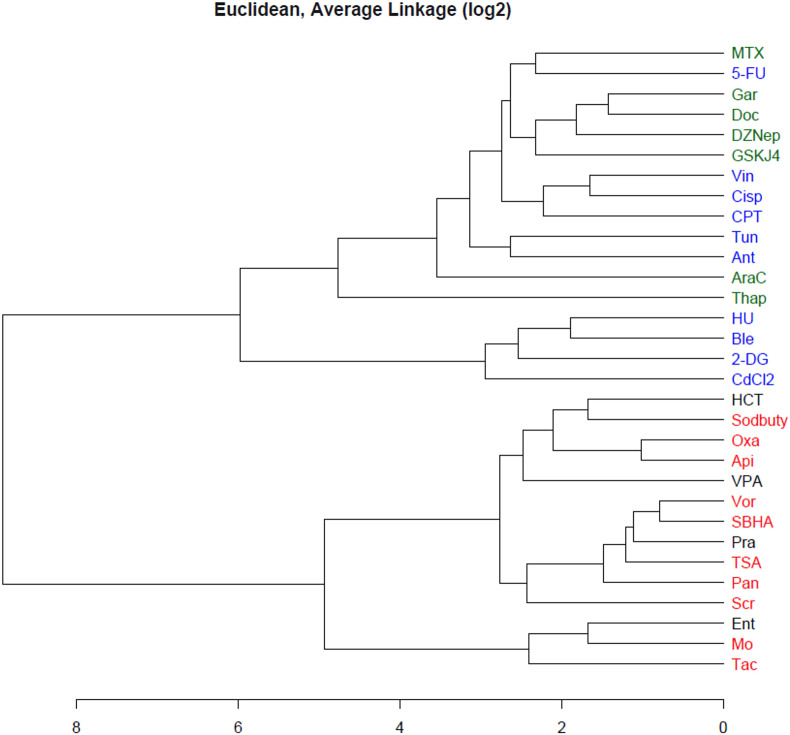
Hierarchical clustering of the 19-gene expression profiles in TK6 cells exposed to the 20 TGx-HDACi reference compounds and 11 external validation compounds for 4 h. Red and blue indicate HDACi reference compounds and non-HDACi reference compounds, respectively. The four HDACi external validation compounds are in black and the seven non-HDACi validation compounds are in green. The two chemical classes formed two separate branches and all 11 validation compounds branched within their respective classes (For interpretation of the references to color in this figure legend, the reader is referred to the web version of this article.).

Figs. 1 and 2 show the principal component analysis (PCA) and hierarchical clustering, respectively, of the 19-gene expression profiles of the 20 reference compounds and the 11 validation compounds. These analyses show two distinct clusters formed by HDACi and non-HDACi classes.

**Fig. 3:**
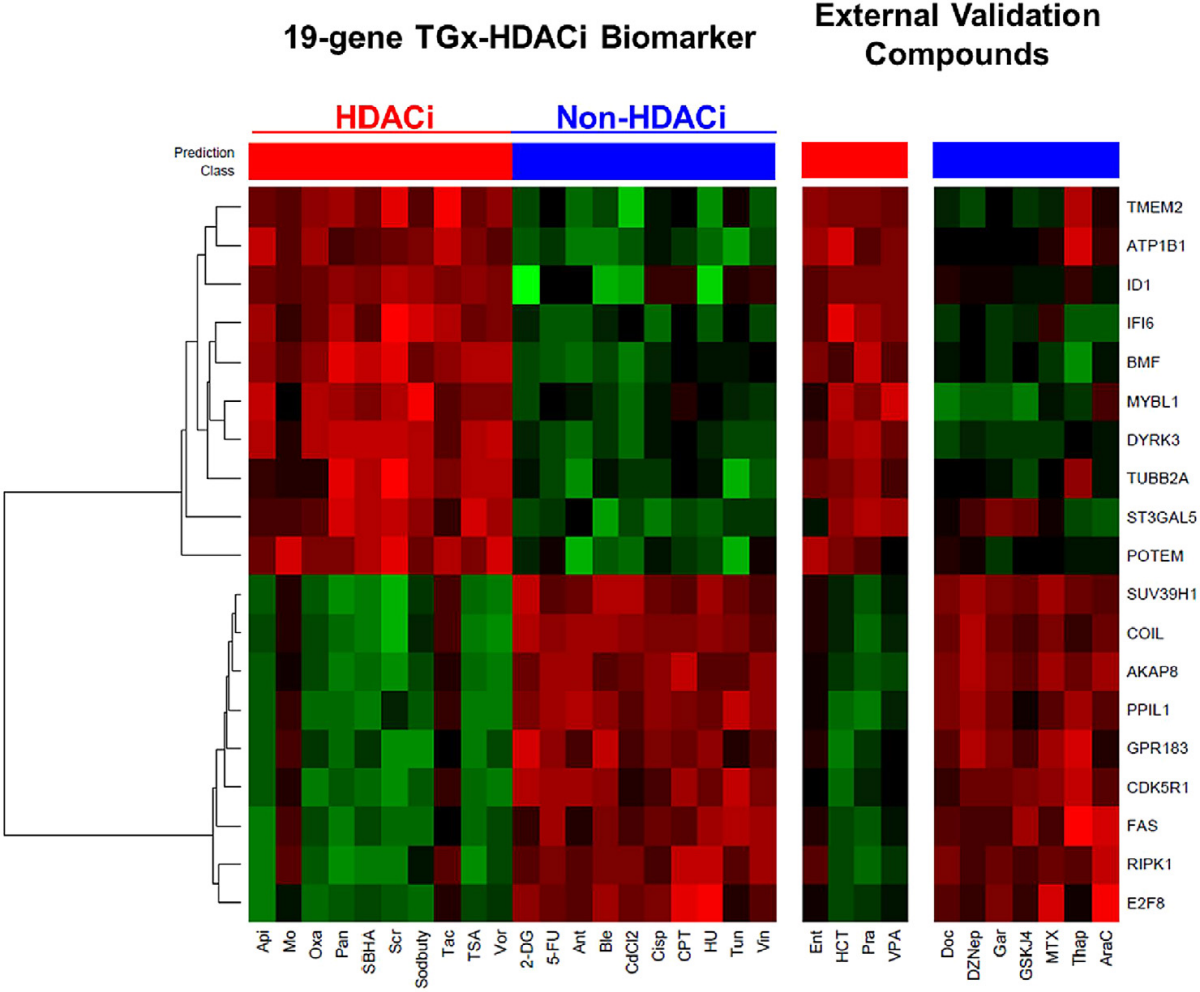
Heatmaps representing the expression level of the 19 genes of TGx-HDACi in TK6 cells exposed to the 20 reference compounds and 11 external validation compounds for 4 h. The chemicals are listed across the *x*-axis. The 19 genes are on the *y*-axis. Red indicates up-regulation and green indicates down-regulation in gene expression. The bars above the heatmaps display the classification calls made by the biomarker in the NSC probability analysis (probability cut-off for HDACi class membership > 90%) and the class to which the chemical belongs. Red indicates HDACi and blue indicates non-HDACi (For interpretation of the references to color in this figure legend, the reader is referred to the web version of this article.).

A graphical depiction of the 19-gene expression profiles with the NSC probability analysis of all chemicals is presented in Fig. 3. The heatmap shows the direction of change in each of the genes in response to the chemical treatment. Each chemical profile was assessed for its probability of membership in the HDACi class based on the expression across the 19 genes. Compounds with above 90% probability of belonging to the HDACi class were classified as such.

## 2 Experimental Design, Materials and Methods

### 2.1 Cell culture

Cell culture and exposure methods are described in Cho et al.. Briefly, TK6 human lymphoblastoid cells (ATCC# CRL-8015; ATCC, Manassas, VA) were cultured in suspension in RPMI1640 medium (Gibco) supplemented with 10% v/v heat-inactivated horse serum (Gibco; New Zealand origin) and 200 µg/mL sodium pyruvate (Gibco), at 37 °C and 5% CO2. The density was maintained between 1 × 10^5^ and 1 × 10^6^ cells/mL in a T75 flask.

### 2.2 Chemical treatment and RNA extraction

TK6 cells were plated in 6-well plates at a density of 4 × 10^5^ – 5 × 10^5^ cells/mL. The cells were exposed for 4 h to one concentration of each of the 20 TGx-HDACi reference compounds (10 HDACi and 10 non-HDACi) and the vehicle solvents, as well as 11 external validation compounds (4 HDACi and 7 non-HDACi), and the vehicle solvents (treatment solution concentration of 1% v/v in media). All chemicals and solvents and the concentrations used in the study are listed in Cho et al. [1].

The concentration selection method is described in detail in Cho et al. [1]. Briefly, in a range finding experiment using qPCR and the 3-(4,5-dimethylthiazol-2-yl)-2,5-diphenyltetrazolium bromide, or MTT, assay for cell viability, these concentrations induced the highest transcriptional responses after 4 h exposures, without reducing cell viability below 60% at the 24 h time point. After 4 h exposures to the selected concentrations, cells were harvested and total RNA was extracted using a RNeasy Mini Kit (Qiagen, Toronto, ON, Canada). The quality of each RNA sample was evaluated using a NanoDrop ND-100 spectrophotometer (Thermo Scientific, Burlington, ON, Canada) and an Agilent 2100 Bioanalyzer or an Agilent TapeStation (Agilent Technologies, Mississauga, ON, Canada). All RNA samples had A260/280 absorbance ratios of ≥ 2.0 and RNA integrity number (RIN) between 7.5 and 10.

### 2.3 Template Oligo-Sequencing library construction

Two 96-sample whole transcriptome TempO-Seq (BioSpyder, Carlsbad, CA) kits were used following the manufacturer's protocol to construct two sets of libraries: one set containing three replicates of each of the 20 reference compound treatments and the corresponding vehicle solvent treatments, and the other containing three replicates of each of the 11 validation compound treatments and the vehicle solvent treatments. Each set of libraries also contained two replicates of water only sample (negative control), human universal reference RNA, and human brain total RNA for quality control and assurance after sequencing. The libraries were sequenced on an Illumina NextSeq 500 using a 75 cycle flow cell.

### 2.4 TempO-Seq data processing

The BCL files were demultiplexed and converted to FASTQ files using bcl2fastq v. 2.20.0.42. Using pete. star. Script_v3.0 (BioSpyder), the reads were aligned and the feature counts specified in a Gene Transfer Format (GTF) file from the aligned reads were extracted. First, low signal (< 0.3%) in the water only samples was confirmed. The two replicates of the human universal reference RNA and the human brain total RNA were compared within each batch and between the two batches of libraries to confirm that data generated from the two sequencing runs were comparable to each other. Both controls had a within-batch correlation of >0.997. When compared between the two batches of libraries, correlation ranged from 0.713 to 0.794.

Boxplots of total mapped reads for each sample and hierarchical clustering of all samples were observed to identify and remove outliers. A dissimilarity cut-off of > 0.2 was applied and three samples (one water vehicle control, one replicate of pracinostat, one replicate of tacedinaline) were removed from the study as a result.

The read counts in all samples were normalized as counts per million (CPM) using R 3.4.1 [3]. The CPM of each chemical treatment sample was normalized to the corresponding vehicle solvent sample. The three replicates of each chemical treatment were averaged to generate the whole transcriptome expression profile of each chemical to be used in HDACi biomarker derivation.

### 2.5 TGx-HDACi biomarker derivation and external validation

The nearest shrunken centroid (NSC) method was applied to the gene expression profiles of the 20 reference compounds to identify a group of genes that can distinguish HDACi from non-HDACi compounds within the reference set [4]. The NSC method was performed using the pamr function in the R statistical environment (www.bioconductor.org). The standard centroids of each of the HDACi and non-HDACi groups were computed by dividing the mean expression level of each gene by its within-class standard deviation.

The centroid shrinkage value was determined based on classification accuracy for the reference compounds in 10-fold cross-validation and the resulting number of genes, with a target range of 50–100 genes. With every increase in the shrinkage value, 10-fold cross validation was performed in which the reference compounds were divided into 10 groups and each of the groups was classified as HDACi/non-HDACi using the remaining nine groups as a classifier in the NSC probability analysis with a probability cut-off of 90%. A cross validation error of 95% was allowed to generate a gene panel in the target range for size. An 81-gene panel with 95% accuracy was identified using a shrinkage value of 3.449.

The 81-gene panel (named the TGx-HDACi biomarker) was used to classify an additional 11 compounds to assess its HDACi classification ability using NSC probability analysis (90% cut-off for the probability of HDACi class membership), principal component analysis (PCA), and hierarchical clustering. Validation compounds that clustered with the HDACi reference compounds were classified as HDACi in the PCA and hierarchical clustering. The PCA was performed using the prcomp in *R* (www.r-project.org) and hierarchical clustering was performed using the hclust function in R using average linkage and Euclidean distance.

### 2.6 Chemical classification using 19 genes of TGx-HDACi

Nineteen genes were identified that are part of both TGx-HDACi and the S1500+ gene panel (Mav et al. 2018). Using the HDACi class centroids of the 19 genes, the 20 reference compounds and the 11 validation compounds were classified as HDACi or non-HDACi in the NSC probability analysis, principal component analysis, and hierarchical clustering to assess its classification ability. Classification calls were made in the same manner as described earlier.

## Ethics Statement

Not applicable.

## CRediT Author Statement

**Eunnara Cho:** Conceptualization, Investigation, Writing – original draft; **Andrew Williams:** Data curation, Formal analyses, Visualization, Writing – review & editing; **Carole Yauk:** Supervision, Funding acquisition, Conceptualization, Writing – review & editing.

## Declaration of Competing Interest

The authors declare that they have no known competing financial interests or personal relationships that could have appeared to influence the work reported in this paper.

## Appendix Supplementary materials

Image, application 1
