# Supplementary material for: A transcriptomic dataset used to derive biomarkers of chemically induced histone deacetylase inhibition (HDACi) in human TK6 cells
Source: Data Brief. 2021 Apr 29;36:107097. doi: 10.1016/j.dib.2021.107097 (PMC8138725; doi:10.1016/j.dib.2021.107097)
Supplement: Supplementary file 1 [file mmc1.docx]

**Supplementary Table 1**

The HDACi and non-HDACi class centroids of the TGx-HDACi biomarker

|  | Centroids | |  |
| --- | --- | --- | --- |
| Gene | **HDACi score** | **Non-HDACi score** | **Standard Deviation** |
| *AKAP8* | -1.758 | -0.086 | 0.930 |
| *AP5S1* | -1.801 | 0.049 | 1.092 |
| *ATP1B1* | 1.268 | -0.645 | 0.898 |
| *BMF* | 3.564 | 0.135 | 1.171 |
| *BNIP1* | -0.899 | 0.464 | 0.845 |
| *C9orf69* | -1.342 | 0.245 | 1.014 |
| *CCDC144NL* | 1.955 | -0.852 | 1.324 |
| *CDK5R1* | -1.542 | 0.043 | 0.954 |
| *CEP68* | 1.659 | -0.508 | 1.230 |
| *COIL* | -1.794 | 0.191 | 1.073 |
| *CPEB4* | 1.680 | -0.370 | 1.171 |
| *DMXL1* | 1.515 | -0.496 | 1.083 |
| *DTWD1* | -1.081 | 0.227 | 0.855 |
| *DYRK3* | 1.769 | -0.031 | 0.860 |
| *E2F8* | -1.497 | 0.226 | 0.985 |
| *EAF2* | 1.234 | -0.058 | 0.844 |
| *ERRFI1* | 1.458 | -0.134 | 1.030 |
| *FAM117A* | 2.189 | -0.458 | 1.663 |
| *FAM217B* | -1.421 | 0.352 | 1.089 |
| *FAS* | -1.317 | 0.353 | 1.007 |
| *GLDC* | 1.428 | -0.493 | 1.046 |
| *GNAZ* | 1.243 | -0.068 | 0.804 |
| *GPR183* | -2.712 | -0.279 | 1.273 |
| *HEY1* | 1.053 | -0.262 | 0.859 |
| *HIP1* | 1.845 | -0.584 | 1.037 |
| *HMCES* | 1.675 | -0.865 | 1.273 |
| *ID1* | 2.287 | -2.029 | 2.607 |
| *IFI6* | 1.506 | 0.044 | 0.896 |
| *IL3RA* | 1.291 | -0.645 | 1.132 |
| *INO80D* | -1.724 | -0.070 | 0.923 |
| *INPP5F* | 1.561 | -0.345 | 0.934 |
| *JADE2* | -1.772 | -0.113 | 1.050 |
| *JARID2* | 1.384 | -0.814 | 1.368 |
| *KLHL42* | -1.368 | 0.393 | 0.962 |
| *LIPH* | 1.399 | -0.819 | 1.289 |
| *MEPCE* | -1.556 | 0.187 | 1.096 |
| *MSL1* | -1.893 | 0.164 | 0.770 |
| *MXI1* | 1.826 | -0.244 | 1.189 |
| *MYBL1* | 2.279 | 0.344 | 1.122 |
| *MYO1E* | 1.036 | -0.438 | 0.930 |
| *NEDD9* | 1.997 | -0.075 | 1.270 |
| *NRROS* | -2.552 | 0.006 | 1.126 |
| *PAPD5* | 1.135 | -0.497 | 1.067 |
| *POTEM* | 1.274 | -0.220 | 0.920 |
| *PPIL1* | -1.709 | 0.078 | 0.978 |
| *PSD3* | 1.452 | -0.629 | 1.150 |
| *PYM1* | -1.623 | 0.121 | 1.072 |
| *RAB11FIP1* | 1.567 | -0.786 | 1.072 |
| *RBM22* | -1.102 | 0.236 | 0.857 |
| *RIPK1* | -1.615 | 0.346 | 1.227 |
| *RNF24* | 1.788 | -0.307 | 1.233 |
| *RWDD3* | -1.143 | 0.377 | 0.909 |
| *SESN3* | 1.062 | -0.254 | 0.833 |
| *SGTB* | 1.396 | -0.314 | 1.072 |
| *SLC12A6* | 1.640 | -0.215 | 1.127 |
| *SMIM14* | 1.115 | -0.512 | 0.923 |
| *SRGAP3* | 1.202 | -1.021 | 1.440 |
| *SRSF4* | -1.466 | 0.175 | 0.869 |
| *ST3GAL5* | 1.257 | -0.414 | 0.906 |
| *SUV39H1* | -1.867 | -0.003 | 1.102 |
| *SWT1* | 1.755 | -0.866 | 1.589 |
| *TBC1D16* | 0.977 | -0.445 | 0.880 |
| *TMCC2* | 2.044 | 0.068 | 0.917 |
| *TMCC3* | 1.611 | -0.814 | 1.206 |
| *TMEM2* | 1.237 | -0.465 | 1.031 |
| *TMEM87A* | -1.488 | -0.003 | 0.960 |
| *TMOD2* | 1.588 | -0.020 | 0.983 |
| *TNFRSF19* | 2.210 | 0.263 | 1.226 |
| *TPCN1* | 1.137 | -0.125 | 0.800 |
| *TRIM8* | -1.414 | 0.128 | 0.912 |
| *TUBB2A* | 1.243 | -0.358 | 1.017 |
| *TULP4* | 0.700 | -0.833 | 0.927 |
| *UGCG* | 1.092 | -0.395 | 0.786 |
| *YARS2* | -1.637 | 0.035 | 0.957 |
| *YES1* | 0.865 | -1.067 | 1.067 |
| *ZBTB1* | -1.484 | 0.041 | 0.953 |
| *ZFX* | -2.146 | -0.262 | 1.177 |
| *ZFY* | -2.664 | -0.257 | 1.380 |
| *ZNF280C* | 0.900 | -0.621 | 0.935 |
| *ZNF282* | -1.101 | 0.010 | 0.727 |
| *ZNF383* | -1.300 | 0.301 | 1.031 |
